# Hematological Profile and Martial Status in Rugby Players during Whole Body Cryostimulation

**DOI:** 10.1371/journal.pone.0055803

**Published:** 2013-02-01

**Authors:** Giovanni Lombardi, Patrizia Lanteri, Simone Porcelli, Clara Mauri, Alessandra Colombini, Dalila Grasso, Viviana Zani, Felice Giulio Bonomi, Gianluca Melegati, Giuseppe Banfi

**Affiliations:** 1 Laboratory of Experimental Biochemistry and Molecular Biology, I.R.C.C.S. Istituto Ortopedico Galeazzi, Milano, Italia; 2 Institute of Bioimaging and Molecular Physiology, National Research Council, Segrate, Milano, Italia; 3 Department of Multifunctional Rehabilitation, I.R.C.C.S. Istituto Ortopedico Galeazzi, Milano, Italia; 4 Centre of Systemic Cryotherapy, Poliambulatorio Bongi, Orzinuovi, Italia; 5 Cardiovascular Department, O.U: Cardiology, Humanitas Gavazzeni, Bergamo, Italia; 6 Department of Biomedical Sciences for Health, University of Milano, Milano, Italia; University of Vermont College of Medicine, United States of America

## Abstract

Cold-based therapies are commonly applied to alleviate pain symptoms secondary to inflammatory diseases, but also to treat injuries or overuse, as done in sports rehabilitation. Whole body cryotherapy, a relatively new form of cold therapy, consists of short whole-body exposure to extremely cold air (−110°C to −140°C). Cryostimulation is gaining wider acceptance as an effective part of physical therapy to accelerate muscle recovery in rugby players. The aim of this study was to evaluate the effect of repeated cryostimulation sessions on the hematological profile and martial status markers in professional rugby players. Twenty-seven professional rugby players received 2 daily cryostimulation treatments for 7 consecutive days. Blood samples were collected before and after administration of the cryotherapic protocol and hematological profiles were obtained. No changes in the leukocyte count or composition were seen. There was a decrease in the values for erythrocytes, hematocrit, hemoglobin and mean corpuscular hemoglobin content, and an increase in mean corpuscular volume and red cell distribution width. Platelet count and mean volume remained unchanged. Serum transferrin and ferritin decreased, while soluble transferrin receptor increased. Serum iron and transferrin saturation were unchanged, as was reticulocyte count, whereas the immature reticulocyte fraction decreased substantially. In conclusion, in this sample of professional rugby players, cryostimulation modified the hematological profile, with a reduction in erythrocyte count and hemoglobinization paralleled by a change in martial status markers.

## Introduction

Cold is commonly applied as a therapeutic physical agent to relieve pain symptoms of inflammatory diseases (rheumatic diseases such as fibromyalgia or rheumatoid arthritis), but it can also be employed to alleviate pain consequent to injuries or overuse, as done in sports medicine. Whole body cryotherapy (WBC), a relatively recent form of cold-based therapy, was introduced in clinical practice about 30 years ago for the symptomatic treatment of rheumatoid arthritis. It consists of short (2–3 minutes) whole-body exposure to extremely cold air (−110°C to −140°C) in a cryochamber [1].

Following demonstration of its benefits (e.g., enhanced muscular recovery after injury or fatigue) in athletes, WBC (in this instance more accurately defined as whole body cryostimulation) has acquired increasing popularity in sports medicine. There is a growing body of evidence for the beneficial effects of WBC: enhancement of cardiovascular function [2], [3]; amelioration of muscular activation and recovery [4]; limitation of sport-induced hemolysis [5]; improvement of the pro- and anti-inflammatory balance [6] and oxidative status [7].

Only one previous study, published by our group in 2008, reported on the hematological response to cryostimulation in rugby players. In detail, treatment was performed once a day for 5 consecutive days. No detrimental effects on hematological parameters were recorded; slight changes in hemoglobin (Hb) and a weak, but significant, decrease in hemoglobinization indexes were found. The authors concluded that the duration of treatment, i.e., the number of cycles, could influence the degree of change [5]. Similar results were obtained in professional field hockey players who received cryotherapic treatment twice daily for 9 consecutive days. Contemporaneous with treatment, the athletes continued training, during which Hb, hematocrit (Ht) and red blood cells (RBCs) were found to decrease in the first part, but then recovered to baseline values by the end of the treatment [1].

To date, no other studies have addressed this issue or evaluated changes in martial status. The noted decrease in hemoglobinization, though slight, could be related either to a direct effect of cryostimulation on bone marrow activity or to the variation in iron availability for cells, also in this case, due to cold therapy.

Studies investigating the effects of training sessions alone on the hematological profile and martial status in rugby players have demonstrated no significant variation in either parameter [8].

Finally, it has recently been argued that WBC constitutes a potential performance-enhancing practice, since techniques that accelerate recovery may be classified as prohibited. WBC treatments, when administered for periods longer or stronger than those previously published, can, in fact, alter biochemical and hematological parameters, pushing them outside the official limits set by sports federations and anti-doping control agencies. In some cases, WBC may be employed as an attempt to mask changes in blood chemistry caused by illicit treatment [1]. In this scenario, studying the effects of WBC has a practical value not only for a variety of physiological and clinical purposes, but also for determining the clinical significance such effects may have in the context of anti-doping settings.

## Materials and Methods

### Ethics Statement

The study was approved by the Reference Ethical Committee (ASL Milano 1). All the subjects involved in this study were informed about the risks and benefits of the treatment protocol and their written consent was obtained for data analysis. The clinical investigations were conducted according to the principles expressed in the Declaration of Helsinki. Of note, cryotherapy sessions and blood drawings were part of the medical treatment and control decided by the team physician. No additional interventions were implemented for the present study.

### Subjects and WBC treatment protocol

Professional rugby players from the Italian National Rugby Team were recruited. All were participating in the 2012 summer training camp held at the end of the competitive season.

The cryotherapy sessions and blood sampling were performed as a part of athletic preparation for the following season and routine medical follow-up in the prevention and/or treatment of muscular injuries which inevitably occur in elite rugby players. The data reported here are the results of an observational protocol.

The study population was 27 males; the mean age was 25.6±3.7 years; the mean weight was 85.9±9.2 kg; and the mean body mass index ([BMI] weight in kilograms divided by the square of the height in meters) was 26.2±1.8 kg/m^2^.

The WBC treatment protocol consisted of 2 daily sessions (one in the morning after the morning training session, and the second in the evening after the afternoon training session) for 7 consecutive days. Each cold-room session consisted of preconditioning for 30 seconds at −60°C then exposure for 3 minutes at −140°C in a special temperature-controlled cryochamber. During the session, the subjects were protected from frostbite with shorts (bathing suit), socks, clogs or shoes, surgical mask, gloves and a cap (or headband) covering the ears. Any sweat was dried before the subject entered the cryochamber, where the air was clear and dry. The subjects were instructed to keep walking while inside the cryochamber, to wriggle their fingers and not to hold their breath. Safety personnel and medical doctors were always on hand.

All of the players were näive to WBC, except for 3 who had received WBC about five years earlier.

### Diet and training

During the summer camp, the athletes trained for a total of 4 hours daily, with anaerobic exercises in the gym every morning and high-intensity discontinuous training on the field every afternoon.

Diet regimen was strictly defined by the team physicians and included an adequate quantity of iron and folates. The diet regimen and training program were similar to those of the 2 weeks preceding the camp. No supplements or medications were administered during the camp or the 2 weeks previously. One subject ingested salicylic acid (1 g/die for 2 days) during the first part of the week to relieve flu symptoms.

### Blood sampling and evaluation of the hematological profile and martial status

Blood samples were collected twice: the first prior to the start of the training camp and the WBC protocol, and the second after the last WBC session. The blood samples were collected in the morning (08:00 a.m.) under standard conditions by antecubital venipuncture on fasting subjects at rest in a sitting position. Evacuated tubes (BD Vacutainer Systems, Becton-Dickinson, Franklin Lakes, NJ) were used for the hematological tests (BD K 2 EDTA 3.5 mL tubes) and 7 mL plain tubes (BD SSTII Advance) for iron status determination. Immediately after blood drawing, the tubes were inverted 10 times and stored in a sealed box at 4 °C until arrival at the laboratory. Hb, Ht, RBC, reticulocyte percentage (Ret%), immature reticulocyte fraction (IRF), absolute and differential counts of white blood cells (leukocyte), platelets (Plt), mean corpuscular volume (MCV), mean corpuscular hemoglobin (MCH), mean corpuscular hemoglobin concentration (MCHC), red cell distribution width (RDW-CV) were performed on a Sysmex XE 2100 (Sysmex, Kobe, Japan). Iron (Fe), transferrin (Tf) and Tf saturation (%) were tested on a Siemens Advia1800 (Siemens, Tarrytown, NY); ferritin was measured on a Siemens Centaur; soluble transferrin receptor (sTfR) was measured on a Siemens BN Prospec (Siemens). Imprecision of hematological tests was <2%, except for Ret% which have an imprecision <4% (e-Check lot 10990810-811-812, Sysmex). Imprecision of chemistry tests was <4.2%.

During the study, the analysers were regularly calibrated and controlled by both internal and external quality control schemes. A day-by-day control of imprecision was performed on the Sysmex instrument by using fresh blood during the study, giving an imprecision <1.6% for Hb, Ht, RBC indices and WBC. All the academic pre-analytical warnings [9] concerning the sample handling and transport, as well as the official anti-doping guidelines, were scrupulously followed [10].

### Statistical analysis

In humans engaging in physical activity, repeated measurement of blood parameters are affected by changes in plasma volume [11]. The percentage of plasma volume change (PV%), eventually due to the intervention, was calculated from the changes in values of hematocrit (Ht) and hemoglobin (Hb) according to the Dill and Costill formula [11] and modified according to Harrison [12], since a change in temperature is applied:
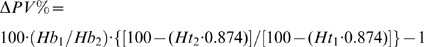



where: Hb_1_ and Hb_2_ were the hemoglobin concentrations before and after the intervention, respectively, while Ht_1_ and Ht_2_ were the hematocrit (in decimal form) before and after the intervention, respectively.

The pre- and post-intervention OFF-score values were calculated as follows:
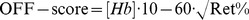



according to the Athlete's Biological Passport guidelines [13], [14].

Statistical analysis was performed using GraphPad Prism v5.0 software (GraphPad Software Inc., LaJolla, CA). In the descriptive analysis, the normally distributed values are expressed as the mean ±SD, and the parametric values are described by median and range (5^th^–95^th^ percentile). Normal distribution of values was assayed with the Kolmogorov-Smirnov normality test. A two-tailed paired t-test for normally distributed values was applied to compare the pre- and post-treatment values, while Wilcoxon's matched pairs test was used for not-normally distributed values. The significance level was set at 0.05.

## Results

### Hematological Profile

Plasma volume changes were not uniform across the study population. The mean ΔPV% was 1.51% between T2 and T1 (second and first blood drawings, respectively), but the range spanned from a minimum of −6.24% to a maximum of 8.75%.

There was no significant change in either the absolute or differential leukocyte counts. In contrast, there were notable changes in the RBC and Hb-related parameters, with a significant decrease in RBCs (from 5.11±0.33×10^12^/L to 4.98±0.27×10^12^/L; p <0.001), Hb concentration (by 3% from 150.6±8.4 g/L to 146.9±6.2 g/L; p <0.001), and Ht (from 45.79±2.41% to 45.20±1.89%) and a significant increase in the distribution width of RBCs (RDW-CV%) (from 13.39±0.42% to 13.09±0.44%; p <0.001).

No change in MCH was observed, while a slight but significant increase was noted for MCV (from 89.78±3.38 fL to 90.83±3.10 fL; (p <0.001), with a consequent decrease in MCHC (from 32.90 (32.10–33.80) g/dL to 32.50 (23.09–33.24) g/dL).

No changes were found in platelet concentration or volume (MPV).

Table 1 reports the changes in the hematological parameters.

**Table 1:** Hematological profile before and after WBC.

	Pre-WBC	Post-WBC	P value
Leu (10^9^/L)	6.10±1.28	5.69±1.58	n.s.
Leukocyte formula			
Neu%	48.41±9.32	47.50±5.09	n.s.
Ly%	39.58±9.01	39.95±5.71	n.s.
Mo%	8.49±1.81	8.69±1.72	n.s.
Eo%	2.50 (1.60−5.74)	2.75 (1.60−7.29)	n.s.
Ba%	0.35 (0.20−0.70)	0.25 (0.10−0.84)	n.s.
Neu (10^9^/L)	2.85 (1.93−4.64)	2.70 (2.10−3.82)	n.s.
Ly (10^9^/L)	2.38±0.71	2.30±0.46	n.s.
Mo (10^9^/L)	0.50 (0.40−0.77)	0.50 (0.30−0.70)	n.s.
Eo (10^9^/L)	0.20 (0.10−0.37)	0.20 (0.10−0.37)	n.s.
Ba (10^9^/L)	0.020 (0.010−0.040)	0.020 (0.003−0.074)	n.s.
RBC (10^12^/L)	5.11±0.33	4.98±0.27	<0.001
Hb (g/L)	150.6±8.4	147.0±6.2	<0.001
Ht%	45.79±2.41	45.20±1.89	<0.01
MCV (fL)	89.78±3.38	90.83±3.10	<0.001
MCH (pg)	29.52±0.97	29.52±0.90	n.s.
MCHC (g/dL)	32.9 (32.1−33.8)	32.5 (23.1−33.2)	<0.001
RDW-CV%	13.09±0.44	13.39±0.42	<0.001
Plt (10^9^/L)	207±37	209±39	n.s.
MPV (fL)	11.15 (10.23−12.07)	11.40 (10.04−12.08)	n.s.

Values are expressed as mean ±SD or median (5^th^–95^th^ percentile). Leu: leukocytes; Neu: neutrophils; Ly: lymphocytes; Mo: monocytes; Eo: eosinophils; Ba: basophils; RBC: red blood cells; Hb: hemoglobin; Ht: hematocrit; MCV: mean corpuscular volume; MCH: mean corpuscular hemoglobin; MCHC: mean corpuscular hemoglobin continent; RDW-CV: red cell distribution width – coefficient of variation; Plt: platelets; MPV: mean platelet volume.

### Martial status

Serum iron remained unchanged (98.74±21.72 mg/dL vs. 92.22 ± 23.45 mg/dL); however, there was a significant, albeit slight, decrease in both transferrin (from 245.33±27.44 mg/dL to 239.74±22.97 mg/dL; p <0.05) and ferritin (from 159.64±74.61 µg/L to 147.71±75.28 µg/L; p <0.05) concentrations.

Transferrin saturation remained substantially unchanged (28.0%, range 19.1–40.9) versus 27%, range, 17.1–37.8), whereas a significant increase was observed in sTfR (from 1.12 mg/L, range, 0.98–1.59 to 1.21 mg/L, range 1.01–1.59).

**Figure 1:**
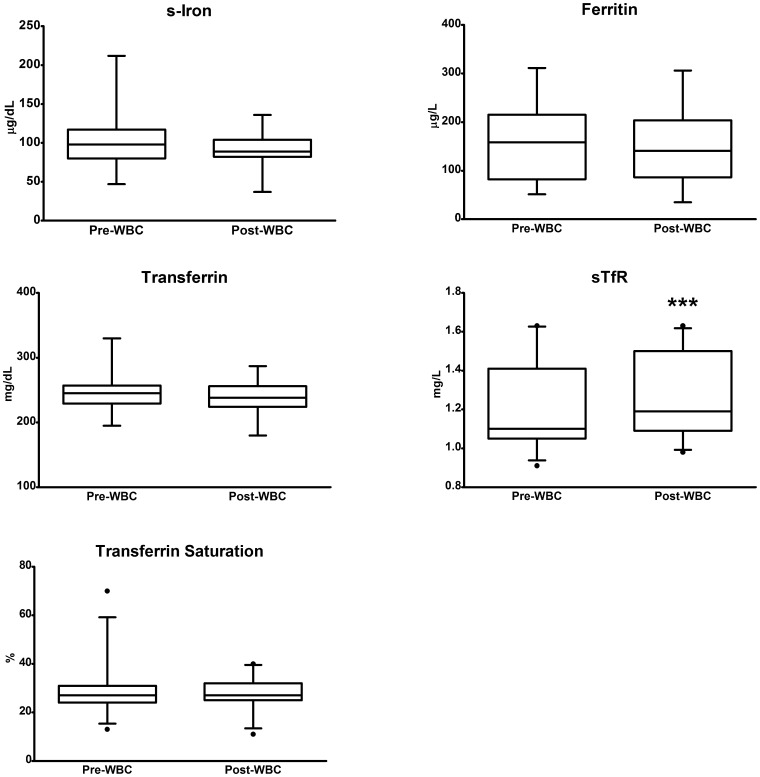
Changes in the levels of the iron-related parameters following whole-body cryostimulation (WBC). The figure shows the levels of a panel of iron-related parameters before and after 14 WBC sessions. The plot represents: mean, standard deviation (box), minimum and maximum (whiskers) for serum iron, ferritin and transferrin; median (box), 5^th^ and 95^th^ percentiles (whiskers) for sTfR and transferrin saturation. Asterisks indicate significant changes (*** p <0.001).

Figure 1 illustrates the changes in the iron-related parameters.

### Reticulocytes, IRF and OFF-score calculation

**Figure 2:**
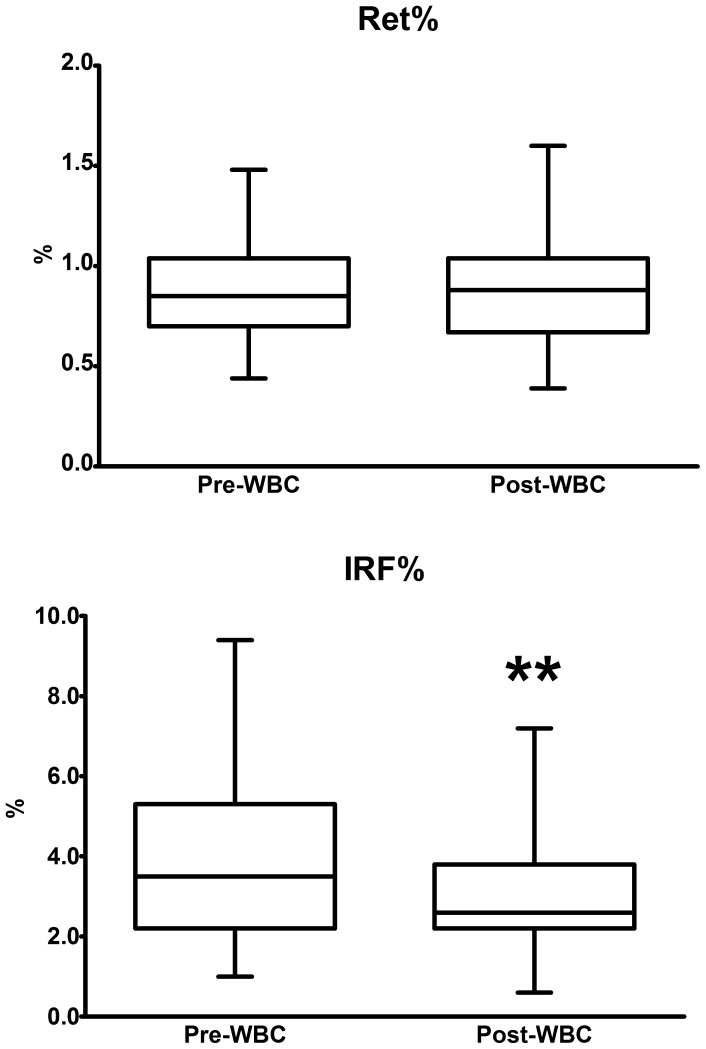
Changes in reticulocyte percentage and immature reticulocyte fraction following WBC. The figure shows the relative counts of Ret and IRF before and after 14 WBC sessions. The box and whiskers plot represents mean, standard deviation (box), minimum and maximum (whiskers). Asterisks indicate significant changes (** p <0.01).

Ret% remained fairly stable (0.86±0.27% before and 0.89±0.28% after WBC), whereas IRF decreased by 27% (from 4.04±2.09% to 2.97±1.39%; p <0.01). The values were within the physiological ranges for both these parameters; a lower post-WBC IRF value (0.6%) was noted in only 1 subject (Fig. 2).

A significant decrease in the OFF-score (from 95.57±10.67 to 90.93±8.91; p <0.01) was observed.

## Discussion

Descriptions of the physiological response to cryotherapy, especially in athletes, who most often undergo such treatment, are very few and they are mainly addressed to evaluate its effects on cardiovascular functions [1].

In a previously published study by our group [5], we reported on the hematological response to WBC in 10 male professional rugby players who received 1 treatment session daily for a total of 5 days. However, a more recent paper by Lubkowska et al. demonstrated the importance of the number and frequency of WBC sessions on physiological parameters in healthy subjects [15]. Following on their findings, we have evaluated the effects of twice daily WBC sessions over 7 days on the hematological profile, and also on the iron status, in a more consistent group of professional rugby players.

Although it was believed that, in both physiotherapy and sports medicine, 10 daily sessions are sufficient to obtain beneficial effects, no solid scientific evidence supports this practice. In a series of recent papers, Lubkowska et al. [3], [7], [15], [16] reported on the effects of various different number of sessions per cycle (10, 15, and 20 daily WBC sessions) on several physiological parameters, including hematological parameters. In one of the studies [3], 25 healthy men were submitted to 15 daily WBC sessions. Despite the different therapeutic frequency compared to our study, a similar downward trend for RBCs, Hb and Ht was found. The main difference between our findings study and those of the previous one [3], resides in the amplitude of the changes, respectively: −2.6% vs. −6.9%, for RBCs; −2.4% vs. −7.5%, for Hb; and −0.59 vs. −0.30%, for Ht. This phenomenon could be imputed to either the difference in the study populations (physically active men vs. elite sportsmen) or the actual level of physical activity (recreational vs. daily professional training). On the other hand, Lubkowska et al. reported an increase in the absolute number of leukocytes after both 10 [7] and 15 [3] daily WBC sessions, whereas we found a downward, though not significant, trend.

In their paper, Banfi et al. [5] demonstrated that the WBC protocol administered to the athletes, associated with light training (3 h per day), and thus comparable to the training protocol applied in the present study, led to a decrease in plasma [Hb] and the indexes of RBC hemoglobinization, MCH and MCHC. Moreover, while Ret% remained unchanged, the mean reticulocyte volume (MRV) and the immature reticulocyte fraction (IRF%) both decreased.

A similar trend was observed in the present study, confirming that the decrease in hemoglobinization could be a peculiar feature of WBC treatment. Indeed, we found a decrease in plasma [Hb] and a contraction of the hematocrit possibly due to the reduction in RBC count. The decrease in RBCs, and subsequently in Ht, is a novel finding in this study since it was not observed in the previous one. This could have been due to the higher frequency of the WBC sessions (2 per day vs. 1 per day) and the longer treatment (7 days vs. 5 days).

The absence of any involvement of the leukocyte compartment suggests that WBC has no effect on the immune system, neither activating nor inhibiting it, which reconfirms that WBC has no detrimental effect on hematological parameters.

The demonstrated lack of any increase in [Hb] following WBC [5], as confirmed by the present study results, argues against considering WBC as an unethical or fraudulent practice or even as a potential tool for masking blood-doping practices.

In a previous study, our group evaluated the effects of training and competition on the hematological profile and iron status in elite rugby players from the same team [8]. When undergoing training sessions, during a camp (defined as the period between the first and the second time-points), with a training protocol comparable to that applied in the present study, none of the subjects showed significant changes in any of the hematological parameters analyzed, whereas, as regards the martial status, only serum iron was seen to decrease significantly. This implies that training does not affect blood physiology in rugby players, [8].

To our knowledge, no previous studies have evaluated the possible effects of WBC treatment on martial status. Its putative effects on iron metabolism could be of particular interest in the application of WBC for hastening recovery. Indeed, iron is a fundamental mineral, and different levels of iron deficiency can affect an athlete, possibly resulting in symptoms. Iron deficiency without anemia may adversely affect athletic performance and can be related to various different causes: poor intake, menstrual losses, gastrointestinal and genitourinary losses due to exercise-induced ischemia or organ movement, foot-strike hemolysis, thermohemolysis, and sweat losses [17]. Besides being a component of the functional core of Hb, iron is also a building block in a number of mitochondrial enzymes involved in energy metabolism, all of which functions are modulated by exercise. Accordingly, tissue iron deficiency has been demonstrated to decrease performance in both animal and human studies, whereas long-term supplementation improves response to exercise [18].

Generally, hematological changes during intense training (decrease in RBC, Hb and Ht) parallel the fall in ferritin concentration, mirroring a depletion of body iron stores [19]. Elite athletes usually display low ferritin levels, although a real iron deficiency might not be really present [20]. It is known that sports-induced inflammation is one of the main causes of iron deficiency in athletes [21] and it is reported that inflammation increases ferritin (even in the presence of iron deficiency anemia) and transferrin levels and reduces sTfR concentrations [22], [23].

In spite of an increase in RDW, no striking instances of iron deficiency were noted in our study population, even though the subjects had just finished the competitive season. Interestingly, following the WBC cycle, a significant decrease in both transferrin and ferritin and an increase in sTfR were evident: these results indirectly confirm the anti-inflammatory effect of WBC since it shifts the levels of these iron status markers in the direction opposite that seen in an inflammatory condition [22], [23]. However, these changes in the iron status markers seem to be addressed towards a reduced iron uptake; this observation is in line with the reduction in hemoglobinization consequent to WBC.

Finally, we remark that the diet regimen and the training protocol applied during the camp were the same as those applied during the 2 weeks preceding the camp. Therefore, the observed changes in the haematological profile and the martial status could be ascribed to cryostimulation.

In conclusion, we found that WBC in elite rugby players induces some changes in the hematological compartment of the body, with a reduction in RBC counts and their hemoglobinization, accompanied by a parallel shift in martial status markers. No effects on reticulocyte counts were noted, while IRF was found to decrease. Finally, we demonstrated that WBC reduces the OFF-score, corroborating the notion that it is unlikely to be considered a doping-assimilable practice.

The main limitation of this study is the lack of a control group. This lack accounts for the difficulty to discriminate the real effects of WBC from the cumulative effects of training and WBC. However, the results reported in previous studies, performed on equivalent populations, provide a base for a possible comparison that could partially overcome this lack. We underline that, unfortunately, this limitation is inevitable since elite athletes train together at the camps and undergo the same interventions. In this regard, it must be remembered that previous similar studies [2], [3], [5], [7], [24] were drawn up in the same way. Nonetheless, case-control studies in elite athletes are needed to confirm these findings.
